# Tracing the sources and spatial distribution of organic carbon in subsoils using a multi-biomarker approach

**DOI:** 10.1038/srep29478

**Published:** 2016-07-06

**Authors:** Gerrit Angst, Stephan John, Carsten W. Mueller, Ingrid Kögel-Knabner, Janet Rethemeyer

**Affiliations:** 1 Chair of Soil Science, Technical University of Munich, Emil-Ramann-Straße 2, D-85354 Freising, Germany; 2 Institute for Geology and Mineralogy, University of Cologne, Zülpicher Straße 49a, D-50674 Cologne, Germany; 3 Institute for Advanced Study, Technical University of Munich, Lichtenbergstraße 2a, D-85748 Garching, Germany

## Abstract

Soil organic carbon (SOC) from aboveground and belowground sources has rarely been differentiated although it may drive SOC turnover and stabilization due to a presumed differing source dependent degradability. It is thus crucial to better identify the location of SOC from different sources for the parameterization of SOC models, especially in the less investigated subsoils. The aim of this study was to spatially assess contributions of organic carbon from aboveground and belowground parts of beech trees to subsoil organic carbon in a Dystric Cambisol. Different sources of SOC were distinguished by solvent-extractable and hydrolysable lipid biomarkers aided by ^14^C analyses of soil compartments <63 μm. We found no effect of the distance to the trees on the investigated parameters. Instead, a vertical zonation of the subsoil was detected. A high contribution of fresh leaf- and root-derived organic carbon to the upper subsoil (leaf- and root-affected zone) indicate that supposedly fast-cycling, leaf-derived SOC may still be of considerable importance below the A-horizon. In the deeper subsoil (root-affected zone), roots were an important source of fresh SOC. Simultaneously, strongly increasing apparent ^14^C ages (3860 yrs BP) indicate considerable contribution of SOC that may be inherited from the Pleistocene parent material.

In recent years, the importance of subsoils for soil organic carbon (SOC) storage and the terrestrial carbon cycles has increasingly been recognized^1^,^2^. Surprisingly, most of the studies on SOC dynamics have been conducted in very shallow subsoils with a median sampling depth of 20 cm^1^, although a significant amount of SOC may be stored well below the first meter of the soil profile^3^. The SOC stored at greater soil depths has been found to generally feature low ^14^C contents (corresponding to high apparent ^14^C ages) that decrease with increasing depth^2^,^4^,^5^. This finding suggests that the SOC stored there is either partly inherited from the parent material or stabilized over longer periods of time^2^,^6^. Because subsoils are commonly unsaturated in organic carbon (OC) and microbial activity has been found to be low^7^,^8^, some authors regarded subsoils as having the potential to sequester additional carbon^6^,^9^. However, the processes and factors that are important to OC stabilization in subsoils still remain poorly investigated^2^.

The main source of SOC is plant-derived organic matter, stemming from either aboveground or belowground plant tissues. The composition, location, and amount of aboveground and belowground organic matter input is substantially different and assumed to drive SOC turnover and stabilization^10^,^11^. Aboveground sources of organic matter are leaf/needle litter and partly tree bark^12^. With ongoing time, the aboveground plant inputs become incorporated into the organic layer and mineral soil via the soil fauna^13^. Belowground inputs are root-derived litter or root exudates^14^ that are directly supplied to the soil *in situ*. Recent studies suggest that SOC from aboveground and belowground sources may highly differ in its degradability^15^. In a litter manipulation experiment, root-derived compounds have been found to be a source of SOC with greater relative stability, whereas aboveground leaf litter was found to be the source of the most actively cycling OC^10^. This points to the importance of unravelling the origin and spatial distribution of SOC as it determines the fate of plant-derived C, either as mineralized CO_2_ or as stabilized SOC^16^. However, there is still considerable debate on the origin of SOC in subsoil, supplied either from roots, or transported down the profile as dissolved or particulate organic matter from aboveground litter and the humus layer^16^,^17^,^18^. Consequently, knowledge on the distribution of SOC from different sources is associated with a high degree of uncertainty. A better empirical basis, identifying soil organic matter constituents from different sources in subsoils is also crucial for modelling deep soil dynamics in order to guide land management practices and climate change mitigation^19^.

In forest soils, the input of SOC from aboveground and belowground vegetation parts may be strongly dependent on the distance to the trees^20^. This spatial dimension has mostly been overlooked, and there have only been a few studies that involved the factor ‘distance’ in their sampling design. The studies performed so far yield no uniform results. For example, in one study, a significant small-scale variability of SOC stocks was found but with no clear relation to the distance from individual beech trees^21^. In another study, an influence of the distance to individual beech trees on the chemical composition of soil organic matter fractions and SOC contents was absent^22^. However, these studies did not differentiate aboveground and belowground sources of SOC. To the best of our knowledge, there has been only one study to date that distinguished plant sources of SOC in a spatially coordinated sampling design^23^. The authors found strong horizontal and vertical gradients in SOC from different plant sources mainly controlled by the rooting zone of individual trees.

An approach to distinguish aboveground and belowground sources involves the analysis of hydrolysable lipid biomarkers that are distinct to either root or shoot plant materials. The biopolymers cutin (leaf-derived) and suberin (root-/bark-derived) fulfil this requirement and have increasingly been used to study the fate of shoot- and root-derived SOC, respectively^23^,^24^,^25^. Besides cutin and suberin, solvent-extractable lipids have been used to investigate the contribution of root-, leaf-, and microbial-derived compounds to soil organic matter^26^,^27^,^28^,^29^. Although solvent-extractable lipids are not necessarily distinct to aboveground and belowground sources of SOC, they can be ascribed to either plant material when concentrations of *n*-alkanes or carboxylic acids highly differ in roots and leaves^30^. The combined use of extractable and hydrolysable lipids has been shown to provide complementary information on vegetation history and soil processes, such as leaching and bioturbation^31^.

The aims of this study were to reveal the contributions to subsoil OC from aboveground and belowground plant sources at increasing soil depths (down to 110 cm) and distances to individual beech trees using solvent-extractable and hydrolysable lipid biomarkers. ^14^C measurements were related to the depth and distance dependent distribution of the biomarkers to identify zones of differently stable SOC.

## Results

### Bulk parameters

Because no horizontal differences in the investigated parameters could be detected, all data are displayed as statistical means ± standard error of the mean (s.e.m.) summarised for all transects and horizontal sampling spots at the respective depth (cf. Statistics and calculations section).

Concentrations of both root biomass and necromass showed significant vertical decreases (p < 0.01) between the densely rooted upper subsoil (0 and 35 cm depths, B horizons) and the deeper subsoil (85 and 110 cm depths, C horizons) (Table 1^22^).

The SOC contents displayed a similar pattern with high contents in the upper subsoil (10 cm: 11.6 ± 1.1g SOC kg^−1^ soil and 35 cm: 5.2 ± 0.5 g SOC kg^−1^ soil) and significantly lower contents at depths of 85 and 110 cm (<0.5 ± 0.1 g SOC kg^−1^ soil; p < 0.01) (Table 1^22^,^32^).

The radiocarbon contents (Table 1) decreased slightly in the upper subsoil at the depths of 10 and 35 cm from 0.988 ± 0.009 fMC (95 ± 75 yrs BP) to 0.905 ± 0.009 fMC (810 ± 80 yrs BP). Below the 35 cm depth, strong decreases were determined, with values of 0.723 ± 0.026 fMC (2650 ± 300 yrs BP) at the 60 cm depth, 0.624 ± 0.028 fMC (3860 + 400 vrs BP) at the 85 cm depth and 0.652 ± 0.064 fMC (3750 ± 810 yrs BP) at the 110 cm depth.

### Lipid biomarkers

#### Solvent-extractable lipid biomarkers

**Figure 1:**
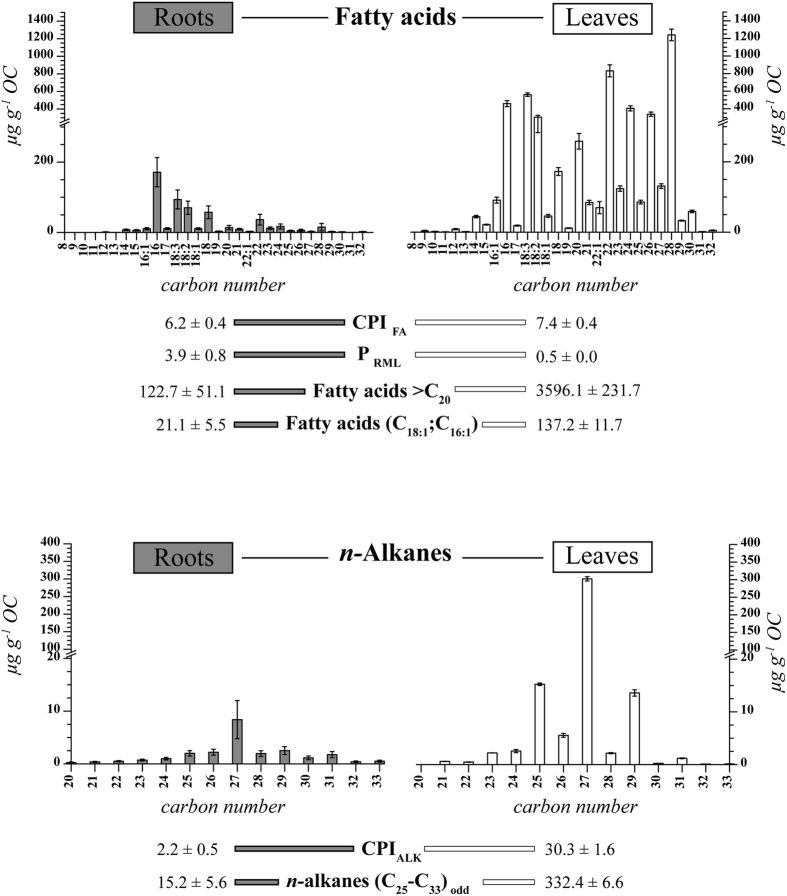
Distribution patterns of the solvent-extractable lipids of the leaf (n = 3) and root (n = 3) material from the study area.

The concentration of solvent-extractable lipids highly differed between roots and leaves. The most prominent differences were observed in the concentrations of the odd-numbered *n-*alkanes C_25_-C_33_ (equation (1), 332.4 ± 6.6 μg g^−1^ OC in leaves and 15.2 ± 5.6 μg g^−1^ OC in roots) and *n*-fatty acids >C_20_ (equation (2), 3596.1 ± 231.7 μg g^−1^ OC in leaves and 122.7 ± 51.1 μg g^−1^ OC in roots), which were several orders of magnitude higher in leaves compared with those in roots (Fig. 1 and Supplementary Table S1). These differences enabled us to develop the P_RML_ ratio as a proxy for root-/microbial-derived SOC in contrast to mainly leaf-derived SOC (cf. Methods section).

**Figure 2:**
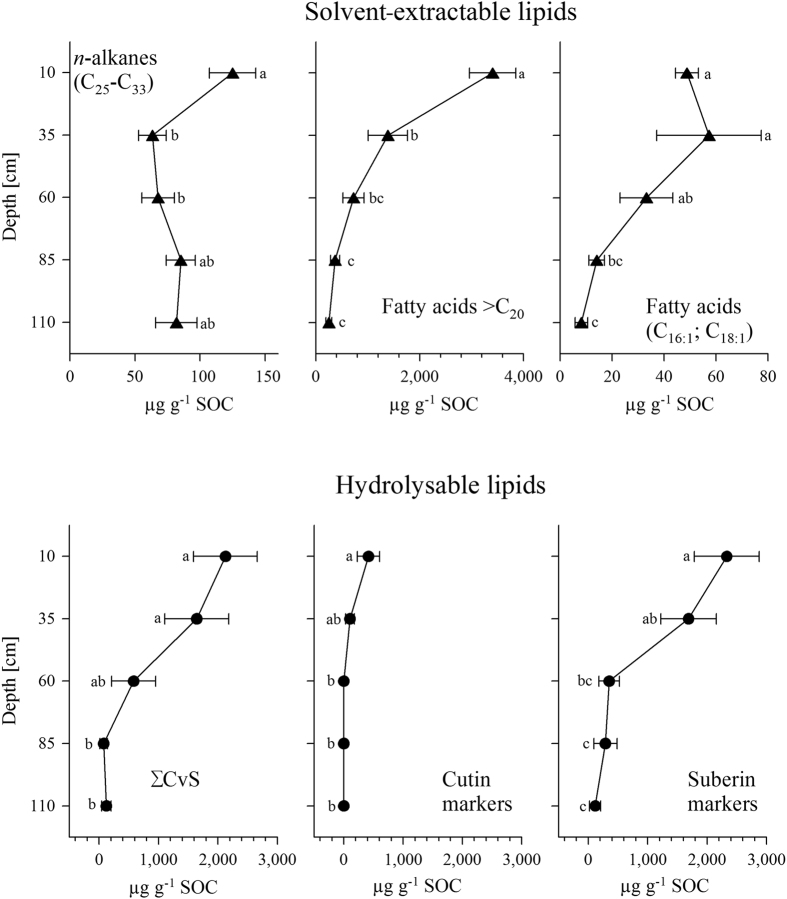
Concentrations of the solvent-extractable and hydrolysable lipid biomarkers (mean of all transects and horizontal sampling spots ± s.e.m.) at different soil depths. Significant differences (*p* < 0.05) are indicated by different letters (a, b, c). n = 9 for each parameter and depth increment.

**Figure 3:**
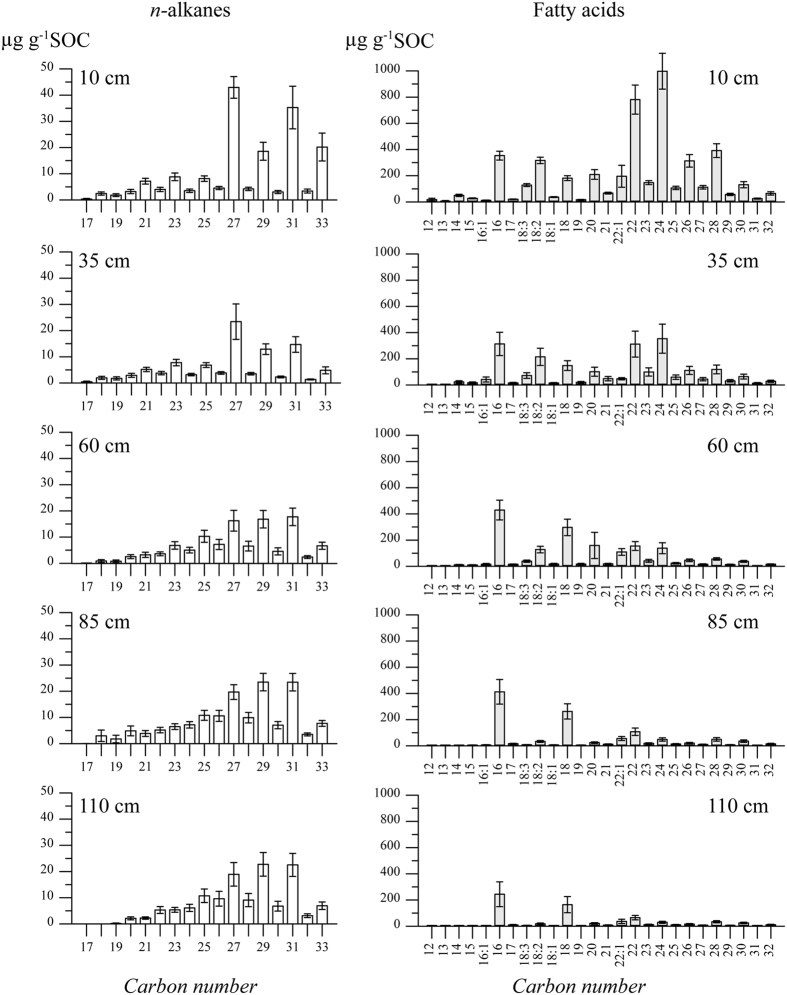
Distribution patterns of the solvent-extractable lipids at different soil depths (n = 9 for each soil depth and lipid type).

In the strongly rooted upper subsoil (10 and 35 cm depths), the contents of plant-derived *n-*alkanes (C_25_-C_33_) decreased significantly (p = 0.02) between the 10 and 35 cm depths and then remained constant with a slightly increasing trend below the 35 cm depth (Fig. 2). The dominant *n*-alkane in the upper subsoil was C_27_. The distribution patterns changed with depth towards longer chain lengths dominating at C_29_ and C_31_ below the 35 cm depth in the deeper subsoil (Fig. 3). In contrast, the plant-derived fatty acids >C_20_ decreased from the 10 to 110 cm depths (Fig. 2). The *n*-fatty acid distribution patterns were strongly dominated by C_22_ and C_24_ (Fig. 3). Below the 35 cm depth, the distribution patterns changed to being dominated by C_16_ and C_18_. The fatty acids mainly derived from microorganisms (C_16:1_; C_18:1_, equation (3)) decreased strongly from 57.3 ± 20.1 μg g^−1^ SOC at the 35 cm depth to 8.2 ± 2.4 μg g^−1^ SOC at the 110 cm depth.

**Figure 4:**
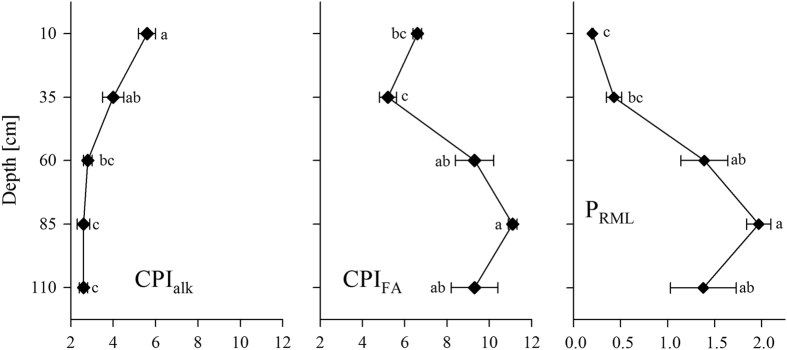
Mean of all transects and horizontal sampling spots ± s.e.m. of the carbon preference index (CPI) of *n*-alkanes (CPI_Alk_) and *n*-fatty acids (CPI_FA_), and the proxy for root-/microbial- vs. leaf-derived SOC (P_RML_). Significant differences (*p* < 0.05) are indicated by different letters (a, b, c). n = 9 for each parameter and depth increment.

The P_RML_ proxy (equation (4), Fig. 4) established in this study showed generally narrow ratios (leaf dominated) in the upper subsoil at the 10 and 35 cm depths in the range from 0.20 ± 0.01 to 0.43 ± 0.08 and wider ratios (root and/or microorganism dominated) at the depths from 60 to 110 cm (1.38 ± 0.35 to 1.97 ± 0.13).

The CPI_Alk_ for *n-*alkanes (equation (5)), as a proxy for the degree of degradation of these lipids, decreased from 5.6 ± 0.4 (10 cm depth) to 2.8 ± 0.2 (60 cm depth) and remained constant below the 60 cm depth (Fig. 4). The low CPI_Alk_ values at the 60 to 110 cm depths were very similar to those found in *n-*alkanes from beech roots (2.2 ± 0.5; Fig. 4). A different trend could be observed in the values of CPI_FA_ (equation (6)) for *n*-fatty acids. This index showed a decreasing trend from the 10 cm depth (6.6 ± 0.2) to the 35 cm depth (5.2 ± 0.4), but strongly increased below the 60 cm depth to a maximum value of 11.1 ± 0.2 (85 cm depth) in the deeper subsoil.

#### Hydrolysable lipid biomarkers

All hydrolysable lipid biomarkers (Supplementary Table S2) showed significant differences (p < 0.01) between the densely rooted upper subsoil (10 and 35 cm depths) and the less rooted deeper subsoil (85 and 110 cm depths) (Fig. 2). The suberin (root markers) and ∑CvS markers (plant markers; equations (8) and (9), see Methods section) decreased from 2127.9 ± 546.9 and 2127.3 ± 535.9 μg g^−1^ SOC at the 10 cm depth to 288.1 ± 195.4 and 78.6 ± 64.0 μg g^−1^ SOC at the 85 cm depth. Notably, the distribution of the suberin markers widely resembled that of the ∑CvS markers. The concentrations of the cutin markers (leaf markers; 414.4 ± 188.6 μg g^−1^ SOC at the 10 cm depth, equation (7)) were substantially lower than that of the suberin and ∑CvS markers, and no cutin markers could be detected in depths greater than 35 cm (Fig. 2).

#### Principle component analysis

**Figure 5:**
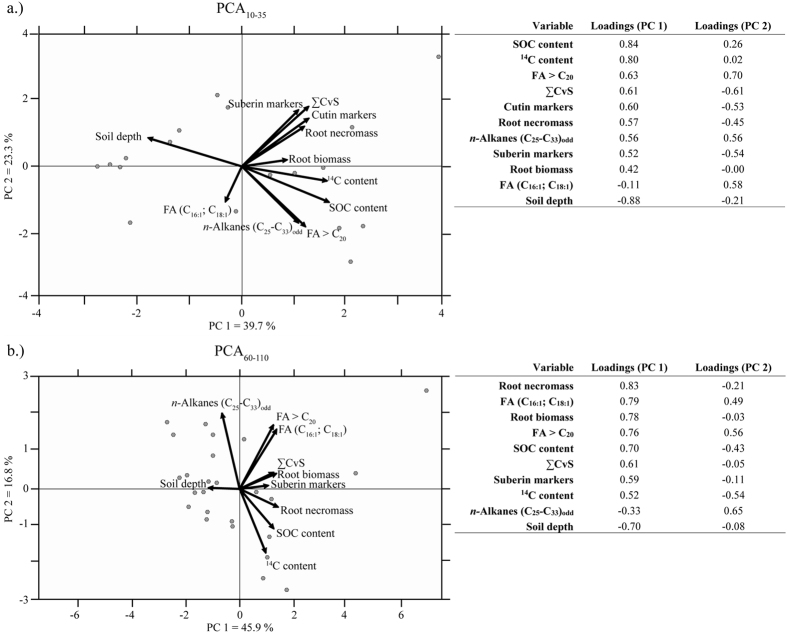
Biplots of the principal component analyses (PCA) for (**a**) the densely rooted upper soil layers (PCA_10–35_, 10–35 cm depths), including 18 data points with 11 variables, and (**b**) the less densely rooted deeper soil layers (PCA_60–110_, 60–110 cm depths), including 27 data points with 10 variables (excluding cutin markers). The loadings (displayed as correlation coefficients) on PC1 and PC2 of the respective PCA are shown in the tables. FA = Fatty acids.

The principle component analysis (PCA) was performed to evaluate the correlation of the lipid biomarkers and the soil parameters in the strongly rooted upper subsoil (PCA_10–35_ for 10–35 cm depths; Fig. 5a) as opposed to the less rooted deeper subsoil (PCA_60–110_ for 60–110 cm depths; Fig. 5b). The first two principle components (PCs) together explained 63% of the variation of the data (PC 1 = 39.7% and PC 2 = 23.3%; Fig. 5a). Principal component 1 was mostly influenced by the SOC and ^14^C contents. Principal component 2 separated the solvent-extractable (negative contribution) from the hydrolysable (positive contribution) lipid biomarkers. The SOC and ^14^C contents were strongly positively correlated with root biomass, fatty acids >C_20_ and the *n-*alkanes (C_25_-C_33_) and to a lesser extent with root necromass and hydrolysable lipid biomarkers identified in this study. Notably, the root necromass and hydrolysable lipid biomarkers plotted together as well as the plant-derived fatty acids >C_20_ and *n-*alkanes (C_25_-C_33_) (Fig. 5a). A negative correlation could be detected between soil depth and all other variables, with the exception of the unsaturated fatty acids (C_16:1_; C_18:1_), which were widely uncorrelated to the investigated biomarkers and soil parameters.

The first two PCs (PC 1 and PC 2) of PCA_60–110_ explained 62.7% of the variability of the dataset (PC 1 = 45.9%, PC 2 = 16.8%; Fig. 5b). Principal component 1 was mainly influenced by the root necromass and biomass and soil depth. Principal component 2 was mostly influenced by the solvent-extractable lipids (*n*-fatty acids and *n-*alkanes) and ^14^C contents. The ∑CvS and suberin markers were positively correlated with the root biomass and were most closely related to the SOC contents, as was the case for the root necromass and ^14^C contents. The analysed solvent-extractable lipids were less correlated with the root necromass and biomass and negatively correlated with the SOC and ^14^C contents. Similar to PCA_10–35_, the soil depth was negatively correlated with most of the investigated parameters.

## Discussion

The concentrations and homologue distribution patterns of the solvent-extractable lipids of the beech leaves, which were dominated by *n*-fatty acids >C_20_ and the C_27_ *n*-alkane, were similar to the results of previous studies that investigated the lipid composition of European beech leaves^33^,^34^. To the best of our knowledge, the solvent-extractable lipid composition of European beech roots has not been reported so far. The composition was dominated by C_16_ and C_18_ homologues for fatty acids and C_27_ for *n*-alkanes (Fig. 1). The most striking characteristic of the solvent-extractable lipids in beech leaves and roots were their highly differing concentrations in *n*-alkanes (C_25_-C_33_) and *n*-fatty acids (>C_20_) (Fig. 1 and Supplementary Table S1). These differences enabled us to (1) infer a leaf source of SOC where concentrations of the *n*-alkanes (C_25_-C_33_) and *n*-fatty acids (>C_20_) in the soil were considerably high and (2) develop a proxy (P_RML_) for the differentiation of leaf and root/microbial sources of SOC.

The concentrations of suberin monomers released from the beech roots and the upper soil layers were in the range of concentrations detected in a study that also investigated soil and plant tissues in a European beech stand^23^. The concentrations of cutin monomers released from the beech leaves in the present study were approximately four times lower than those observed in the aforementioned study. This result may be due to the extraction of leaf litter in the present study in contrast to the extraction of fresh leaves. However, the comparison of such data from different studies is complicated because the lipid composition may change with the life span or morphology of leaves and roots^35^.

The statistical analysis of the data revealed no influence of the distance from the trees on the solvent-extractable and hydrolysable lipids as well as on the ^14^C contents of the SOC (cf. section 2.6). Instead, a pronounced vertical gradient could be detected with the largest decrease of the investigated parameters between the densely rooted upper subsoil (10 and 35 cm, corresponding to B horizons) and the less densely rooted deeper subsoil (60–110 cm, corresponding to C horizons). These results reflect the findings of previous studies that investigated the chemical composition and distribution of soil organic matter fractions in the same transects and ^14^C contents in one of the transects^5^,^22^. The authors did not find any horizontal trend but a similar vertical gradient down to the 110 cm depth as was observed in the present study. The authors hypothesised that OC inputs by roots likely played a dominant role for the observed patterns because of a dense and even rooting of the upper subsoil (10 and 35 cm depths) and considerably higher SOC contents of rhizosphere than that of bulk soil. This hypothesis could be confirmed and expanded by the source identification of SOC in the present study.

The subsoil in the present study could be differentiated into two vertical zones, a ‘leaf- and root-affected zone’ and a ‘root-affected zone’.

The SOC in the leaf- and root-affected zone, corresponding to the upper subsoil (B horizons at 10 and 35 cm depths), was composed of a mixture of fresh leaf- and root-derived compounds, evidenced by a positive correlation of SOC contents with the solvent-extractable and hydrolysable lipids and root biomass and necromass (Fig. 5a). The relatively high ^14^C contents at the 10 cm depth (0.988 ± 0.009 fMC; 95 ± 75 yrs BP) support the presence of SOC from fresh sources (Table 1). The declining ^14^C contents at the 35 cm depth (0.905 ± 0.009 fMC; 810 ± 80 yrs BP) indicate an increasing contribution of SOC derived from a relatively old source and/or decreasing concentrations of fresh plant-derived SOC. In addition to root-derived SOC, the strong correlation of the long-chain *n*-alkanes (C_25_-C_33_) and *n*-fatty acids (>C_20_) with SOC contents indicate the importance of leaf-derived SOC in the upper subsoil. This finding is supported by the low values of P_RML_ (Fig. 4). The low correlations of cutin markers with SOC contents may be explained by an already advanced stage of decomposition of the leaf litter, which is also reflected in the low concentrations of cutin monomers released from the extracted leaves. Comparably low concentrations of the predominantly microbial-derived fatty acids (C_16:1_, C_18:1_) that were uncorrelated with the SOC contents suggest a low contribution of microbial-derived compared with plant-derived SOC (Fig. 5a). Furthermore, the microbial-derived fatty acids were uncorrelated to the soil depth, indicating a ubiquitous occurrence of microbes in the upper subsoil.

The SOC in the root-affected zone, corresponding to the deeper subsoil (60–110 cm depths, C horizons), was composed of relatively high amounts of old SOC (minimum values of 0.624 ± 0.028 fMC; 3860 ± 400 yrs BP) and smaller proportions of younger, mainly root-derived SOC. The dominance of root- in contrast to leaf-derived SOC was clearly implied by the presence of suberin along with the absence of cutin markers. Furthermore, dead fine roots were found to be no older than 20 yrs^36^,^37^,^38^. Thus, the positive correlation of the fine root necromass with SOC and ^14^C contents (Fig. 5b) indicates that the root necromass was a major source of fresh SOC at greater soil depths (60–110 cm depths). Strongly increasing CPI_FA_ values from depths of 35 to 60 cm and below (Fig. 4) also indicate the presence of fresh SOC in the C horizons. The high values of P_RML_ (Fig. 4) indicate the dominance of root-/microbial-derived C_16_ and C_18_ fatty acids compared with mostly leaf-derived >C_20_ fatty acids. The slightly hydrophilic short-chain fatty acids (C_16_ and C_18_) are either translocated from the upper soil layers or are produced *in situ* by microorganisms or roots^39^. In this regard, microbial-derived SOC appeared to be of minor importance because the concentrations of the microbial-derived fatty acids (C_16:1_, C_18:1_) were low and strongly correlated with the plant-derived fatty acids >C_20_, indicating that the former were rather derived from plant material from which trace amounts of these acids were released (Fig. 1). This finding questions the assumption of subsoil OC being enriched in microbial-derived SOC^40^. Surprisingly, the root biomass was almost uncorrelated to the SOC contents, indicating that root exudates appeared to be of minor importance, probably because of their higher lability in soils^41^,^42^. The weaker correlation of the root necromass to the suberin markers in the depth range of 60 to 110 cm compared with the upper subsoil (10 and 35 cm depths) may be explained by a higher stage of degradation of the root necromass, which was most likely more depleted in suberin monomers. The high correlation of suberin and ∑CvS markers in the depths of 60 to 110 cm indicates that the latter were most probably also root-derived. Thus, our results support the notion of Rasse *et al.*^16^ that fresh SOC inputs to the deeper subsoil are mainly root-derived.

However, a considerable amount of the SOC located at the depths of 60 to 110 cm was very old and probably partly inherited from the parent material. Long-chain *n*-alkanes may contribute to the older SOC pool at greater depth and were found to be relatively stable against decomposition^43^,^44^. These long-chain *n*-alkanes may thus be an important indicator for past vegetation^45^,^46^,^47^. The constant or slightly increasing concentrations of *n*-alkanes with increasing soil depth (Fig. 2), which were also observed by others^29^,^44^, and the strong negative correlation of *n*-alkanes with the ^14^C contents support their contribution to old SOC. Similar results were reported by others, who found an accumulation of aliphatics with soil depth that were likely not derived from the current vegetation^48^. This inference is further corroborated by very low values of CPI_Alk_ (Fig. 4), indicating a high degree of degradation and, in turn, a relatively high residence time of the *n*-alkanes in the investigated subsoil. Notably, the CPI_Alk_ in the depths of 60 to 110 cm was highly similar to the CPI_Alk_ observed for the beech roots (Supplementary Table S1), suggesting that the CPI_Alk_ in soil may also reflect a more recent input of root-derived SOC^49^. Generally, the CPI_Alk_ values calculated from long-chain *n*-alkanes must be interpreted with caution because they may vary strongly in different plant species from 0.039 to 99^50^. Another indication for SOC that is not derived from the present vegetation is provided by a change in the distribution patterns of *n*-alkanes in the depths of 60 to 110 cm from a dominance of beech-derived C_27_ *n*-alkanes to a dominance of C_29_ and C_31_ *n*-alkanes (Fig. 3). However, compound-specific radiocarbon analyses of *n-*alkanes are required to undoubtedly prove the assumption that these lipids were considerably old. Synthetically, all the data for the deeper subsoil indicate that some of the SOC located at these depths likely originated from an old source and may potentially be inherited from the parent material. Similarly, other authors stated that the very old apparent ^14^C ages of some soils may reflect the dilution of inherent geogenic carbon with younger SOC^2^. Although this SOC has low contents in the deeper subsoil, it considerably contributes to SOC stocks^22^ and is thus highly relevant for the C cycle and SOC modelling.

Our results have important implications for C allocation in subsoils. Considerable amounts of leaf-derived SOC (presence of cutin markers and low P_RML_ values) were still found in the B horizons of the soil profiles (down to 35 cm depth). This finding indicates that translocation of organic matter has occurred, even at the 35 cm depth in the subsoil. Because cutin is characterised by a low water solubility^51^ a translocation as dissolved OC is unlikely. This finding is surprising because the soil conditions are rather unfavourable for soil fauna (e.g. low pH 3.4–4.5; cf. section 2.1) in the investigated Cambisol. Likewise, translocation may occur as fine particulate organic matter in this sandy and thus highly permeable soil^2^,^18^. The absence of cutin markers in the deeper subsoil (60–110 cm depth) indicates that incorporation of cutin no longer occurs at that depth. Although not directly monitored, our data enabled us to obtain evidence on processes that may be important for the translocation of considerable amounts of leaf-derived SOC into subsoils. In this regard, some authors proposed sequestering SOC in subsoils by planting deep rooting plant species that would allocate root-derived SOC to deep soil layers^3^,^6^. European beech may develop a deep rooting system^20^, and the amount of root biomass and necromass may still be considerably high at soil depths greater than 0.6 m^52^,^53^. Our results do not confirm these hypotheses, but indicate that the recent tree vegetation influences the SOC mainly in the uppermost subsoil horizons (down to the 35 cm depth). The deeper subsoil receives inputs of root-derived organic carbon, but at the same time we find very high apparent ^14^C ages below the 35 cm depth, pointing to a contribution of geogenic C. Our results indicate that the allocation of SOC into deep soil layers cannot be accomplished by simply establishing typical deep rooting plant species, but that site-specific factors may essentially control the spatial dimension of the rooting system.

In summary, we identified lipid biomarkers specific to European beech that enabled us to trace SOC from leaf, root and microbial sources at different soil depths and distances from individual trees. The distribution of lipid biomarkers was not influenced by the distance from individual trees but by vertically stratified inputs of leaf- and root-derived SOC. Accordingly, we distinguished two vertical zones. (1) The root- and leaf-affected zone (10 and 35 cm depth; B horizons) was composed of fresh root- and shoot-derived SOC, indicating that contributions of supposedly fast cycling, leaf-derived SOC may be still important well below the A horizons of a soil. (2) The root-affected zone (60 to 110 cm depth; ICv and IICv horizons) was composed of fresh root-derived SOC, with an important contribution of relatively old SOC with high apparent ^14^C ages (up to 3860 yrs. BP). This old SOC was potentially inherited from the parent material or stabilized over thousands of years and has to be considered as an important contributor to the SOC pool in deep subsoils. These results point to a decelerated decomposition of SOC in the deeper subsoil. Future studies should focus on input pathways of SOC from different sources to help elucidate the evolution of SOC distribution patterns such as those observed in the present study, which may help to guide forest management practices and advance soil C modelling.

## Methods

### Study area and soil sampling

The study was performed at the Grinderwald, a managed, even-aged European beech forest (*Fagus sylvatica* L.) established in 1916, located northwest of Hannover (52°34′22″N, 9°18′51″E), Germany. The predominant soil type was an acidic (pH 3.4–4.5), sandy (77.3% sand, 18.4% silt and 4.4% clay) Dystric Cambisol^54^ developed from sandy glacio-fluvial deposits (Saale glacial), the humus form was moder. The phyllosilicate mineralogy was characterised by the presence of chlorite, mixed-layer minerals, kaolinite and illite, whereas smectites and carbonates were absent. A more detailed description of the study area is given elsewhere^22^.

Three 3.15 m long and 2.00 m deep transects were dug, each starting at the stem base of a mature beech tree. The direction of each transect was chosen such that the stem base of neighbouring trees was not reached to track the influence of a single tree on the spatial distribution of selected soil properties. Composite soil samples (each ~1 kg) were taken next to the tree (0 cm), at an intermediate distance from the tree (135 cm), and far from the tree (270 cm) down to a depth of 110 cm (starting at 10 cm depth with 25 cm depth increments, n = 45). The first vertical sampling spot was set to 10 cm depth (Bsw horizon) to ensure a regular sampling along the grid that is unbiased by varying topsoil thicknesses. This study thus exclusively investigated subsoil samples. In addition, leaf litter (n = 3) and roots (n = 3) of European beech were randomly collected from each transect. The soil samples were air-dried and sieved to <2 mm, and the litter and root samples were freeze dried and finely ground. All samples were subjected to a sequential extraction procedure to release solvent-extractable and hydrolysable lipids. Data regarding root biomass, root necromass and SOC contents were partly derived from previous studies at the same site^22^,^32^. Twelve data points regarding root biomass and necromass were supplied by Kristina Kirfel (Albrecht von Haller Institute for Plant Sciences, Georg-August-Universität Göttingen, Germany).

### SOC analysis

Carbon measurements of all soil samples were performed using an elemental analyzer (EuroVector, Milan, Italy) via dry combustion. An aliquot of 1–2 mg of each sample was ground and used for analysis. All measurements were performed in duplicate. Because carbonates were absent from the study area^22^, all carbon contents were equal to the organic carbon contents.

### Radiocarbon analysis

Because the SOC content of the sand fraction was very low (≤0.3 g kg^−1^;^18^), this fraction was removed by dry sieving (mesh size of 63 μm). All samples were processed using a modified protocol published earlier^55^. Briefly, potentially present inorganic carbon was removed by extraction with 0.5% HCl. The suspension was placed in a drying oven for one hour at 60 °C and then left overnight at room temperature. The hydrochloric acid was removed by washing with Milli-Q water to pH 5. The samples were dried at 60 °C and subsequently graphitized with H_2_ over an iron catalyst. The radiocarbon contents of the samples were then measured on a 6 MV Tandetron AMS (HVE, Netherlands) at the University of Cologne. The results of the ^14^C measurements were reported as fraction modern carbon (fMC) and as apparent conventional ^14^C ages in years before present (yrs BP), related to 1950.

### Sequential liquid extraction procedure

#### Analysis of the solvent-extractable lipids

Lipids were extracted from ~20–50 g of bulk soil and 1.0–1.5 g of beech leaf and root material using accelerated solvent extraction (Dionex ASE 350, USA) with dichloromethane:methanol (9:1, 100 bar, 120 °C, 20 min). The extracts were saponified with methanolic KOH (0.5 M) and then separated into a neutral and an acid fraction by liquid–liquid phase separation (water:dichloromethane). The *n*-alkanes were separated from the dichlormethane phase by eluting with hexane using column chromatography (activated SiO_2_; mesh size 60 μm). After acidification with concentrated HCl, the acid fraction was derivatised using methanolic HCl (95:5). Fatty acid methyl esters (FAMEs) were separated and purified over a SiO_2_–Na_2_SO_4_ column with dichloromethane:hexane (2:1).

The *n-*alkanes and FAMEs were measured using a gas chromatograph equipped with a flame ionisation detector (GC-FID, 5890 series II plus, Hewlett Packard, USA equipped with DB-5MS column 50 m and 5 m pre-column, 0.2 mm ID, 0.33 μm df). Lipid identification and quantification was performed using external standard mixtures.

#### Analysis of the hydrolysable lipids

After pre-extraction of the solvent-extractable lipids, the soil/plant residues were subjected to alkaline hydrolysis to release hydrolysable lipids. The samples, 10 g of soil and 0.5 g of plant material, were saponified with methanolic KOH in teflon lined bombs at 100 °C for 3 hours. The extracts were processed, qualified and quantified using GC/MS, following the procedure described elsewhere^56^. The amounts of aliphatic acids were normalised to the OC content of the respective sample (stated as μg g^−1^ OC).

### Identification of lipid biomarkers for distinguishing aboveground, belowground and microbial sources of SOC

#### Solvent-extractable lipid biomarkers

The vegetation markers in this study were selected according to their occurrence in the analysed beech leaves and roots (Fig. 1, Supplementary Table S1) and previously published biomarkers^25^.

Waxes derived from higher plants are commonly identified by large abundances of long-chain, odd-numbered *n-*alkane homologues C_21_ to C_33_ and long-chain *n*-fatty acids >C_20_^57^,^58^. These compounds were also found to be the most abundant compounds in the beech leaves and roots of the present study (Fig. 1, Supplementary Table S1). Notably, the concentrations of the odd-numbered *n-*alkanes C_25_-C_33_ and *n*-fatty acids C_20_-C_32_ were several orders of magnitude higher in leaves compared with those in roots (Fig. 1). Similar results were obtained by others for *n*-alkanes in the roots and leaves of different plant species^30^. We assume that considerably high concentrations of the mentioned lipids (*n*-alkanes C_25_-C_33_ and *n*-fatty acids C_20_-C_32_) in soil are indicative of SOC being mainly derived from leaves:


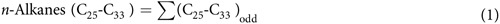


and


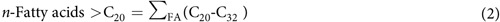


The *n*-fatty acids extracted from beech leaves and roots (Fig. 1) showed not only large differences in the concentrations but also in the distribution patterns of the homologues. The beech leaves were dominated by *n-*fatty acids >C_20_ (equation 2), whereas short-chain *n-*fatty acids C_14_-C_18_ were the most abundant compounds in beech roots, dominated by C_16_ (Fig. 1). We thus used the ratio of short-chain *n-*fatty acids (C_14_-C_18_, derived from roots and/or microorganisms) to long-chain *n-*fatty acids (>C_20_, dominant in leaves in the present study) expressed by the proxy termed P_RML_ (root-/microbial- vs. leaf-derived SOC) to differentiate SOC derived from roots and/or microorganisms in relation to SOC derived from leaves:


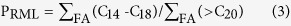


Only trace amounts of mono-unsaturated fatty acids C_16:1_ and C_18:1_ could be detected in the leaves and roots of the present study. Thus, these unsaturated compounds were used as indicators of microbial-derived SOC according to the findings of a previous study^26^:


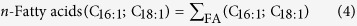


The degradation of plant material leads to decreasing abundances of odd-numbered *n-*alkanes and decreasing even-numbered *n-*fatty acids. This can be identified by the carbon preference index (CPI)^59^, which reflects the odd-over-even and the even-over-odd predominance of *n*-alkanes and fatty acids, as given below, respectively.


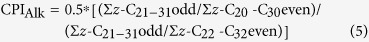



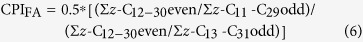


The equations were slightly modified with z being the number of carbon atoms^60^. High CPI values (>10) reflect the input of mainly fresh SOC, and low CPI values (≪10) indicate the degradation of SOC^59^.

#### Hydrolysable lipid biomarkers

Leaves and roots were characterised by different abundances and chain lengths of *n*-carboxylic, ω-hydroxy alkanoic, α,ω-alkanedioic and mid-chain-substituted hydroxy alkanoic acids mainly derived from cutin and suberin (Supplementary Table S2). Because specific cutin- and suberin-derived monomers were found to decompose at similar rates^56^,^61^, we used the sum of the respective monomers in soil to evaluate the contribution of aboveground vs. belowground SOC.

The 8,9,10,ω-dihydroxy hexadecanoic acids (subsumed under x,ω-C_16_) were used as markers for leaf-derived SOC as they were not released from roots and correspond to previously suggested cutin biomarkers^23^,^35^,^56^.


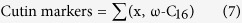


The ω-hydroxy alkanoic acids with a chain length of C_20_, C_22_ and C_24_ (ω-C_20_, ω-C_22_ and ω-C_24_) were used as markers for root-derived SOC as they were not released from leaves and correspond to previously suggested suberin biomarkers^23^,^56^. The α,ω-octadecanedioic acid (C_18_ DA), usually present in both cutin and suberin^25^, was not detected in leaves and thus was added to the specific root markers in this study:


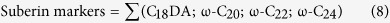


The sum of the unspecific (part of cutin and suberin) monomers, i.e. ω-hydroxy hexadecanoic acid (ω-C_16_), α,ω-hexadecanedioic acid (C_16_ DA) and 9,10,ω-hydroxy octadecanoic acid (9,10,ω-C_18_), was used as a marker for plant-derived SOC (referred to as ∑CvS):


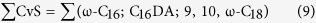


#### Statistics and calculations

Statistical means and s.e.m were calculated using Microsoft Excel 2013 (Microsoft, Redmond, WA, USA). All other statistics (significant if p < 0.05) were computed using the R 3.0.3 software for Windows^62^. The data were analysed to identify significant differences among the three different transects, including the horizontal (0, 135 and 270 cm distances) and vertical (10–110 cm depths). First, the data were tested for normality and homoscedasticity using the Shapiro–Wilk and Bartlett test, respectively. Depending on the outcomes of the tests, significant differences were then evaluated using the one-way analysis of variance (ANOVA) or the Kruskal–Wallis test. The Tukey honestly significant difference (HSD) and Dunn’s test were applied as post-hoc tests. In a previous study, significant differences between the transects regarding the SOC contents, root biomass and necromass were not detected^22^. The same was found for the ^14^C contents and the solvent-extractable and hydrolysable lipids in this study. Thus, we regarded the transects as being replicates. Subsequent analyses among the sampling spots revealed that there were also no significant differences between the horizontal sampling intervals at the respective depths. We therefore present our data summarised as one depth function for each parameter (mean ± s.e.m.). Based on the results of previous studies^22^,^32^, two principle component analyses (PCAs) were performed to separately investigate the strongly rooted upper subsoil (PCA_10–35_, 10–35 cm depths, corresponding to B horizons (Table 1)) and the less densely rooted deeper subsoil (PCA_60–110_, 60–110 cm depths, including the ICv and IICv horizons (Table 1)). The dataset of PCA_10–35_ included 18 data points with 11 variables, whereas the dataset of PCA_60–110_ included 27 data points with 10 variables because cutin markers were not detected in the 60 to 110 cm depths. All variables were standardised (centred and scaled). The PCA was conducted using PAST 3.06 for Windows^63^.

## Additional Information

**How to cite this article**: Angst, G. *et al.* Tracing the sources and spatial distribution of organic carbon in subsoils using a multi-biomarker approach. *Sci. Rep.* **6**, 29478; doi: 10.1038/srep29478 (2016).

## Supplementary Material

Supplementary Information

## Figures and Tables

**Table 1:** Soil parameters (mean of all transects and horizontal sampling spots ± s.e.m.) at different soil depths: SOC contents, root biomass and necromass, ^14^C contents and apparent ^14^C ages.

Depth (cm)	Soil horizon	SOC content (g kg^−1^)	Root biomass (kg m^−3^)	Root necromass (kg m^−3^)	^14^C content (fMC)	^14^C age (yrs BP)
10	Bsw	11.6 ± 0.4a	0.94 ± 0.30a	1.09 ± 0.10a	0.988 ± 0.009a	95 ± 75a
35	Bw	5.2 ± 0.5ab	0.53 ± 0.07ab	0.79 ± 0.19a	0.905 ± 0.009a	810 ± 80a
60	I Cv	1.3 ± 0.3bc	0.18 ± 0.09bc	0.13 ± 0.02ab	0.723 ± 0.027b	2650 ± 300b
85	II Cv	0.5 ± 0.0c	0.01 ± 0.01c	0.00 ± 0.00b	0.624 ± 0.028b	3860 ± 400b
110	II Cv	0.4 ± 0.0c	0.03 ± 0.02c	0.03 ± 0.03b	0.652 ± 0.064b	3750 ± 810b

Significant differences (*p* < 0.05) are indicated by different letters (a–c). n = 9 for each parameter and depth increment.
