# Supplementary material for: Tracing the sources and spatial distribution of organic carbon in subsoils using a multi-biomarker approach
Source: Sci Rep. 2016 Jul 6;6:29478. doi: 10.1038/srep29478 (PMC4933938; doi:10.1038/srep29478)
Supplement: Supplementary Information [file srep29478-s1.pdf]

Supplementary tables

Tracing the sources and spatial distribution of organic carbon in subsoils using a multi-biomarker approach.

Gerrit Angst, Stephan John, Carsten W. Mueller, Ingrid Kögel-Knabner, Janet Rethemeyer

Supplementary table S1. Solvent-extractable lipid concentrations (mean ± SE) of *n*-alkanes and *n*-fatty acids released from the beech leaf (n=3) and root (n=3) material from the study area.

**Solvent-extractable lipids**						
	**Leaves** [µg g^{-1} OC]			**Roots** [µg g^{-1} OC]		
***n*-alkanes**						
Eicosan (n-C_{20})	n.d.			0.2	±	0.1
Heneicosan (n-C_{21})	0.6	±	0.0	0.4	±	0.1
Docosan (n-C_{22})	0.5	±	0.0	0.5	±	0.1
Tricosan (n-C_{23})	2.2	±	0.0	0.7	±	0.2
Tetracosan (n-C_{24})	2.6	±	0.3	1.0	±	0.2
Pentacosan (n-C_{25})	15.2	±	0.2	2.0	±	0.5
Hexacosan (n-C_{26})	5.6	±	0.4	2.2	±	0.6
Heptacosan (n-C_{27})	302.3	±	6.2	8.4	±	3.6
Octacosan (n-C_{28})	2.2	±	0.1	2.0	±	0.5
Nonacosan (n-C_{29})	13.6	±	0.6	2.5	±	0.7
Triacontan (n-C_{30})	0.2	±	0.0	1.1	±	0.4
Hentriacontan (n-C_{31})	1.2	±	0.1	1.8	±	0.6
Dotriacontan (n-C_{32})	0.1	±	0.0	0.4	±	0.1
Tritriacontan (n-C_{33})	0.1	±	0.0	0.5	±	0.2
Sum n-alkanes	346.4	±	8.0	23.8	±	8.0
CPI_{alk}	30.3	±	1.6	2.2	±	0.5
***n*-fatty acids**						
Nonanoic acid (n-$C_{9:0}$)	3.5	±	1.9	n.d.		
Decanoic acid (n-$C_{10:0}$)	2.0	±	0.7	n.d.		
Undecanoic acid (n-$C_{11:0}$)	0.9	±	0.3	n.d.		
Dodecanoic acid (n-$C_{12:0}$)	8.8	±	1.6	1.1	±	0.7
Tridecanoic acid (n-$C_{13:0}$)	1.6	±	0.1	0.2	±	0.1
Tetradecanoic acid (n-$C_{14:0}$)	44.1	±	3.3	7.5	±	2.0
Pentadecanoic acid (n-$C_{15:0}$)	21.5	±	1.0	6.2	±	1.9
Hexadecenoic acid (n-$C_{16:1}$)	91.0	±	8.5	10.5	±	3.1
Hexadecanoic acid (n-$C_{16:0}$)	458.1	±	33.3	171.3	±	41.5
Heptadecanoic acid (n-$C_{17:0}$)	18.7	±	1.5	11.0	±	2.4
Octatridecenoic acid (n-$C_{18:3}$)	560.3	±	19.5	93.8	±	26.9
Octadodecenoic acid (n-$C_{18:2}$)	304.1	±	20.9	70.3	±	18.8
Octadecenoic acid (n-$C_{18:1}$)	46.2	±	4.4	10.7	±	2.6
Octadecanoic acid (n-$C_{18:0}$)	172.6	±	11.2	57.3	±	18.1
Nonadecanoic acid (n-$C_{19:0}$)	11.6	±	1.0	3.1	±	0.7
Ecosanoic acid (n-$C_{20:0}$)	258.4	±	22.2	13.8	±	6.0
Heneicosanoic acid (n-$C_{21:0}$)	84.4	±	6.3	9.1	±	2.4
Docosenoic acid (n-$C_{22:1}$)	69.7	±	17.3	3.1	±	0.2
Docosanoic acid (n-$C_{22:0}$)	831.0	±	68.8	36.4	±	15.2
Tricosanoic acid (n-$C_{23:0}$)	123.8	±	7.5	12.0	±	3.8
Tetracosanoic acid (n-$C_{24:0}$)	404.4	±	28.9	16.9	±	7.1
Pentacosanoic acid (n-$C_{25:0}$)	85.7	±	5.3	4.5	±	1.7
Hexacosanoic acid (n-$C_{26:0}$)	338.1	±	24.3	5.9	±	3.2
Heptacosanoic acid (n-$C_{27:0}$)	131.2	±	6.5	2.5	±	1.3
Octacosanoic acid (n-$C_{28:0}$)	1240.0	±	65.9	15.1	±	10.4
Nonacosanoic acid (n-$C_{29:0}$)	33.0	±	1.4	2.3	±	1.0
Tricontanoic acid (n-$C_{30:0}$)	59.1	±	3.8	1.7	±	0.4
Hentriacontanoic acid (n-$C_{31:0}$)	1.7	±	0.2	0.5	±	0.0
Dotriacontanoic acid (n-$C_{32:0}$)	5.1	±	1.1	2.1	±	0.7
Sum n-fatty acids	5410.8	±	368.8	568.6	±	172.4
CPI_{FA}	7.4	±	0.1	6.2	±	0.4

Supplementary table S2. Hydrolysable lipid concentrations (mean ± SE) of alkanoic acids released from the beech leaf (n=3) and root (n=3) material from the study area. Specific cutin monomers are marked with a dashed frame, specific suberin monomers are bold-framed, and non-specific monomers (part of cutin and suberin) are gray-shaded.

**Hydrolysable lipids**						
	**Leaves** [µg g^{-1} OC]			**Roots** [µg g^{-1} OC]		
**n-alkanoic acids**						
Tetradecanoic acid (n-$C_{14:0}$)	20.3	±	4.6	n.d.		
Hexadecanoic acid (n-$C_{16:0}$)	121.9	±	27.8	104.8	±	35.1
Octadecenoic acid (n-$C_{18:1}$)	3.9	±	0.8	5.6	±	2.0
Octadecenoic acid (n-$C_{18:2}$)	4.5	±	2.0	463.0	±	295.4
Octadecanoic acid (n-$C_{18:0}$)	26.5	±	3.4	24.8	±	7.5
Eicosanoic acid (n-$C_{20:0}$)	12.0	±	2.4	n.d.		
Docosanoic acid (n-$C_{22:0}$)	39.7	±	10.3	n.d.		
Tetracosanoic acid (n-$C_{24:0}$)	31.0	±	6.0	n.d.		
Sum n-Carboxylic acids	*259.9*	±	*57.3*	598.1	±	340.0
**ω-hydroxy alkanoic acids**						
ω-Hydroxyhexadecanoic (ω-$C_{16:0}$)	310.5	±	66.5	1607.1	±	944.2
ω-Hydroxyoctadecenoic acid (ω-$C_{18:1}$)	n.d.			141.3	±	92.2
ω-Hydroxyeicosanoic acid (ω-$C_{20:0}$)	n.d.			1389.9	±	777.6
ω-Hydroxydocosanoic acid (ω-$C_{22:0}$)	n.d.			2727.0	±	1513.3
ω-Hydroxytetracosanoic acid (ω-$C_{24:0}$)	n.d.			77.0	±	35.5
Sum ω-hydroxy alkanoic acids	*310.5*	±	*66.5*	*5942.2*	±	*3362.8*
**α,ω-alkanedioic acids**						
α,ω-Heptadioic acid (C_7 DA)	n.d.			14.7	±	3.5
α,ω-Octadioic acid (C_8 DA)	42.8	±	6.9	44.2	±	12.3
α,ω-Nonadioic acid (C_9 DA)	106.6	±	17.7	65.2	±	17.3
α,ω-Decanedioic acid (C_{10} DA)	17.1	±	1.7	n.d.		
α,ω-Undecanedioic acid (C_{11} DA)	10.1	±	0.7	7.8	±	1.7
α,ω-Dodecanedioic acid (C_{12} DA)	94.6	±	24.4	591.8	±	369.1
α,ω-Hexadecanedioic acid (C_{16} DA)	61.5	±	20.2	559.0	±	290.9
α,ω-Octadecanedioic acid (C_{18} DA)	n.d.			91.4	±	53.5
Sum α,ω-alkanedioic acids	332.7	±	71.6	1374.1	±	748.4
**mid-chain substituted hydroxy and epoxy alkonic acids**						
x,ω-Dihydroxyhexadecanoic acid (x,ω-OHC_{16})	95.9	±	41.8	n.d.		
9,10,18-Trihydroxyoctadecanoic acid (9,10,18-OHC_{18})	26.5	±	9.8	28.8	±	3.8
Sum mid-chain substituted hydroxy alkonic acids	*122.3*	±	*51.6*	*28.8*	±	*3.8*
Sum of extracted aliphatic acids	*1025.5*	±	*180.5*	*7943.2*	±	*4455.0*
